# Three‐step procedure for preparation of pure *Bacillus altitudinis* ribonuclease

**DOI:** 10.1002/2211-5463.12023

**Published:** 2016-01-19

**Authors:** Elena Dudkina, Vera Ulyanova, Raihan Shah Mahmud, Vera Khodzhaeva, Linh Dao, Valentina Vershinina, Alexei Kolpakov, Olga Ilinskaya

**Affiliations:** ^1^ Institute of Fundamental Medicine and BiologyKazan Federal (Volga‐Region) UniversityRussia

**Keywords:** *Bacillus altitudinis*, balnase, binase, homogeneous, purification, ribonuclease, substitution

## Abstract

Ribonucleases are considered as promising tools for anticancer treatment due to their selective cytotoxicity against tumor cells. We investigated a new RNase from *Bacillus altitudinis* termed BALNASE (*B*. *altitudinis* RNase). Balnase is a close homolog of the well‐known cytotoxic binase, differing by only one amino acid residue: nonpolar hydrophobic alanine at position 106 in the balnase molecule is replaced by a polar uncharged threonine in binase. The most exciting question is how the physico‐chemical properties and biological effects of RNase might be changed by A106T substitution. Here, we have developed a chromatography‐based rapid and modern technique for the purification of this new RNase which allowed us to get a protein sample of high quality with specific activity of 1.2 × 10^6^ units in preparative amounts, suitable for further investigation of its biological properties.

**Abbreviations**
Balnase: ribonuclease from *Bacillus altitudinis*
Binase: ribonuclease from *Bacillus pumilus*
FPLC: fast protein liquid chromatography
RNase: ribonuclease

Enzymes involved in RNA metabolism have attracted the attention of many researchers over the years. The most intriguing properties of ribonucleases (RNases) are their selective antitumor effect and antiviral activity 1, 2 and 3. Among cytotoxic ribonucleases, the enzymes of bacterial origin are considered as more promising therapeutics for anticancer treatment than eukaryotic ones due to their ability to evade mammalian ribonuclease inhibitor 4 and 5.

Currently, RNases from different organisms were isolated and purified to homogeneity 6, 7, 8, 9 and 10. The isolation procedures included certain laborious steps based on principals of ion‐exchange chromatography 11 and 6. Another approach for the production of these proteins deals with the application of recombinant *Escherichia coli* species as producers 7, 8, 9 and 10. However, it is known that the purification of recombinant proteins from *E. coli* often results in unfolded/misfolded proteins, especially for heterologous proteins that require longer times and/or molecular chaperones to fold correctly 12. Moreover, the cell wall of gram‐negative bacteria contains a high amount of endotoxins. Therefore, the isolation and purification of RNases from wild‐type strains is considered more favorable for their future application in medicine than recombinant techniques using *E. coli*.

In the beginning of 1990s, Dementiev *et al*. 6 isolated the RNase from *B. thuringiensis* var. *subtoxicus* B‐388. The primary structure of this RNase had a very high level of similarity with binase, RNase from *B. pumilus* (former name *B. intermedius* 13, 14). Amino acid sequencing revealed only one substitution in *B. thuringiensis* RNase as compared to binase. Ala(106) in balnase was replaced by Thr(106) in binase.

*Bacillus thuringiensis* RNase consists of 109 amino acid residues and has a molecular mass of 12 182 Da. The enzyme is a basic protein with an isoelectric point (*pI)* value of 9.5. The thermostability of the protein was found to be up to 53.7 °C 6, 15. The technique of RNase purification suggested by Dementiev *et al*. 15 included different laborious steps of ion‐exchange chromatography. The purity of the protein sample was checked only by SDS/PAGE 15.

During the last decade, the molecular properties and biological effects of binase were investigated. We have shown that binase possesses selective cytotoxicity against lung carcinoma and ovarian cancer cells 16 and 17. It was revealed that the expression of oncogenes *kit, ras*,* AML1‐ETO* and *FLT3* determines a selective susceptibility of transformed fibroblasts and leukemic cancer cells to binase 1 and 18, 19, 20. Earlier, we compared binase with its “molecular twin” barnase, the RNase from *B. amyloliquefaciens,* which possesses 85% homology with binase 14. Actually, the RNase from *B. thuringiensis* is the natural homolog of binase, differing by one amino acid residue only. The most interesting question is how the physico‐chemical properties and biological effects of RNase could be changed by a single substitution of polar uncharged threonine for nonpolar hydrophobic alanine in its molecule. Therefore, we have to isolate and purify the RNase from *B. thuringiensis* for future comparison with well‐studied binase.

However, none of the sequenced *B. thuringiensis* genomes contain RNase genes homologous to binase. Using mass spectrometry and sequencing techniques, we have shown that the taxonomic state of *B. thuringiensis* var.* subtoxicus* B‐388 is not accurate and the strain should be renamed as *B. altitudinis* B‐388 21.

Here, we carried out the purification of new RNase BALNASE (*B*. *altitudinis* RNase) according to an updated technique, which we have elaborated for homogeneous protein preparation. The pure preparation of balnase possesses specific activity of 1.2 × 10^6^ units. We elaborated the simple three‐step method of its routine purification with a total 40% yield of the enzyme (by activity). Our results allowed us to get a protein sample of high quality for further investigation of its biological properties.

## Materials and methods

### Growth conditions

*Bacillus altitudinis* B‐388 (former *B. thuringiensis* var.* subtoxicus* strain number B‐388 in the All‐Russian Collection of Microorganisms ‐ VKM, Russia) was grown on the complex phosphate deficient LP medium (low‐phosphate peptone, 2.0%, glucose, 1.0%, CaCl_2_, 0.01%, MgSO_4_ × 7H_2_O, 0.03%, NaCl, 0.3%, MnSO_4_, 0.01%, pH 8.5) with shaking (200 rpm) at 37 °C.

### Enzyme isolation

Industrially manufactured binase was obtained from the Institute of Organic Syntesis. Riga. The first steps of protein isolation from *B. altitudinis* B‐388 were performed using gravity flow chromatography. Culture fluid (500 mL) was collected after 28 h of bacteria cultivation (at 37 °C, 200 rpm), which corresponded to the maximum accumulation of the enzyme in the medium, and acidified by glacial acetic acid to pH 5.0. The cells were removed at 6000 ***g*** for 30 min at 4 °C. Supernatant was diluted twice with sterile water and flowed through the column (50 × 200 mm) packed with DEAE‐cellulose (Servacel, Germany), equilibrated with 10 mm Na acetate buffer (pH 5.0). After that, suspension (1500 mL) was concentrated on the column with phosphocellulose P‐11 (50 × 200 mm) (Whatman, England) equilibrated with 10 mm Na acetate buffer (pH 5.0) and washed with the same buffer until A_280_ of the eluate reached 0.05. Then, the system was equilibrated with the 20 mm Na phosphate buffer (pH 7.0) to reach pH 7.0 in the column. Proteins were eluted in 200 mm Na phosphate buffer (pH 7.0) with the flow rate of 2 mL·min^−1^. Elution fractions of 5 mL were collected. The quality of protein fractions were checked by measuring the levels of catalytic activity (A_260_) and protein yield (A_280_). Both of the sorbents (DEAE‐cellulose and phosphocellulose P‐11) were prepared as described in the Whatman's manual. Protein samples were concentrated and desalted using Spin‐X UF concentrator.

Subsequent step of enzyme purification was performed using Biologic DuoFlow FPLC system (BioRad, Hercules, CA, USA) on the UNOS_6_ (12 × 53 mm) (BioRad) column equilibrated with 10 mm Na phosphate buffer (pH 7.0). 20 mg of the proteins were loaded on the column. Proteins were eluted with a linear gradient of 0–1.0 m NaCl with the flow rate of 2 mL·min^−1^.

To improve the resolution of enzyme peaks we performed chromatography with step‐up profiles. All conditions were retained, but the time for every stage of chromatography was increased. The quality of protein fractions were checked by measuring the levels of catalytic activity (A_260_) and protein yield (A_280_).

*Specific activity* was calculated as the ratio of enzyme activity to the amount of protein.

*Degree of purification* is the ratio of the specific activity calculated after each purification step to the specific activity of the initial extract. The degree of purification in the culture fluid was taken to be 1.

*Yield* determines the catalytic activity retained after each purification step as a percentage of the activity in the culture fluid. The catalytic activity in the culture fluid was taken to be 100%.

### Catalytic activity

The catalytic activity of *B. altitudinis* RNase balnase was measured against high molecular weight yeast RNA as described in 22. One unit was defined as the amount of enzyme that increases the extinction of acid‐soluble products of RNA hydrolysis at 260 nm per min at 37 °C. The activity was measured in buffer containing 250 mm Tris‐HCl, pH 8.5.

### SDS/PAGE and immunoblotting

Proteins were separated by SDS/PAGE 23. Separating gel contained 15% acrylamide, stacking gel contained 6% acrylamide. Protein samples were suspended in 4x sample buffer (0.5 m Tris‐HCl, 10% glycerol, 10% SDS, 0.01% bromophenol blue, 14.5 m β‐mercaptoethanol, pH 6.8). Electrophoresis was performed at 150V for 60 min. Proteins were stained with Coomassie R‐250.

After separation by SDS/PAGE proteins were transferred to a nitrocellulose membrane by semi‐dry electroblotting. For the detection of proteins anti‐binase antibodies were isolated from rabbit blood as described earlier 24. The concentration of anti‐binase antibodies was 1 : 2000. Visualization of protein bands corresponding to RNases was performed using anti‐rabbit IgG‐POD secondary antibodies (Sigma‐Aldrich, St. Louis, MO, USA) and the LumiLight detection system (Roche Diagnostics, Basel, Switzerland).

### Zymography

To estimate RNase activity of proteins in the gel we performed zymography analysis as described in 25. Proteins were separated in 15% polyacrylamide gel with 0.1% SDS (SDS/PAGE) 23. The resolving gel contained RNA from Torula yeast (Sigma‐Aldrich, USA) as a substrate at final concentration of 7 mg·mL^−1^. Then, the gel was washed with buffer I (10 mm Tris‐HCl, 20% isopropanol, pH 7.5) for 10 min to remove SDS and then proteins were refolded by consequent incubation for 10 min in 10 mm Tris‐HCl, pH 7.5 and in 100 mm Tris‐HCl, pH 7.5. The gel was stained for 10 min with 0.2% toluidine blue (Sigma‐Aldrich, USA).

### Mass spectrometry

Proteins were excised from the SDS/PAGE (1.5 × 1.5 mm), washed with acetonitrile and 200 mm NH_4_HCO_3_ (mixed in ratio of 1 : 1) and digested with trypsin (Promega, Fitchburg, WI, USA) overnight at 37 °C. The peptides extraction was carried out using 0.1% trifluoroacetic acid. LC‐MS/MS analysis was performed using HPLC LC‐MS/MS system (Bruker, Billerica, MA, USA). The samples were loaded in 0.1% formic acid and eluted at a flow rate of 300 nL·min^−1^ in a linear gradient of acetonitrile (5–60%) during 50 min.

The homogeneity of the proteins in solution was checked using MALDI TOF/TOF system by the same scheme except for protein extraction from the gel.

### Modelling

Putative models of proteins were generated using I‐Tasser server (http://zhanglab.ccmb.med.umich.edu/I-TASSER/). Comparison of physico‐chemical properties of proteins was performed using protparam tool (http://web.expasy.org/protparam/).

## Results

### Time‐course of secreted RNase production by *Bacillus altitudinis*

**Figure 1:**
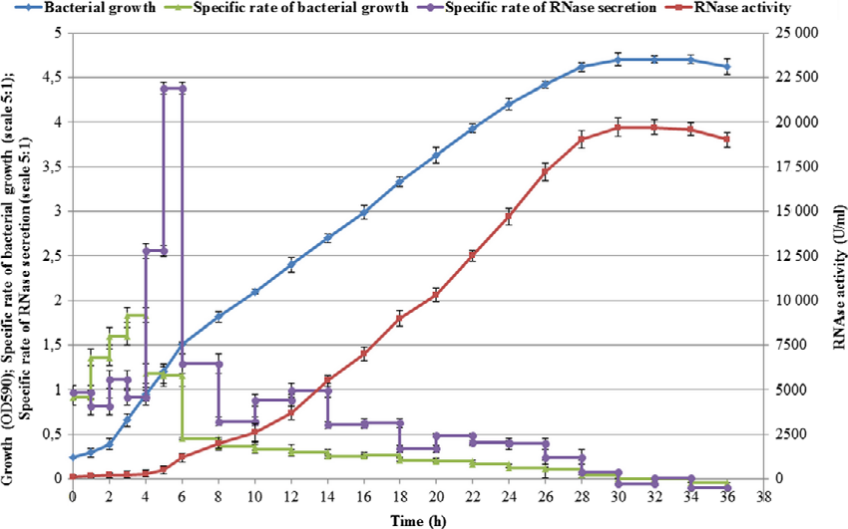
Time‐course of *Bacillus altitudinis* B‐388 growth and RNase production. The specific growth rate was calculated as Δln OD590/Δt and RNase production as Δln A260/Δt, where t is time. Scale 5 : 1 shows values in five times greater than real ones.

To find the period of maximal RNase accumulation we investigated the dynamics of RNase secretion during *B. altitudinis* growth (Fig. 1). It was shown earlier that inorganic phosphates inhibit the production of guanyl‐preferring RNases 26 and 27. On the basis of this data, we performed experiments using a phosphate deficient medium for *B. altitudinis* growth. Usually, secreted RNases of bacilli are accumulated in the culture medium at the stationary phase of bacterial growth 27. For *B. altitudinis*, the stationary phase began after 28 h of cultivation and finished at 34 h of bacterial growth. The maximum enzyme accumulation measured by catalytic activity peaked at 28–34 h (Fig. 1).

### Three‐step‐process of balnase purification

**Figure 2:**
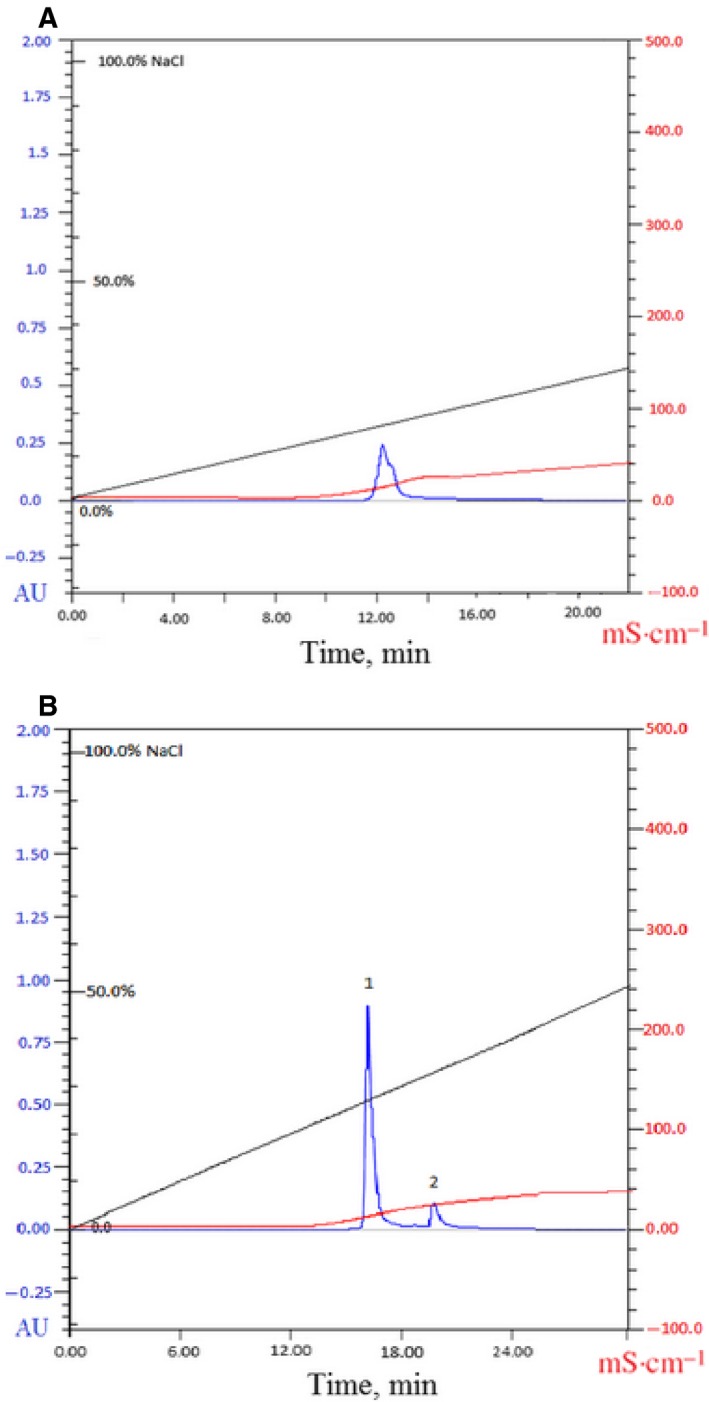
Elution of balnase from UNOS6 column in a linear gradient of 0–1.0 m NaCl using FPLC Biologic DuoFlow system. (A) chromatography on UNOS6 column, (B) chromatography on UNOS6 column with step‐up profiles; 1 – elution profile of balnase, 2 – elution profile of hypothetical protein HQ51_17865. AU – absorbance units, mS·cm^−1^ – conductivity.

**Figure 3:**
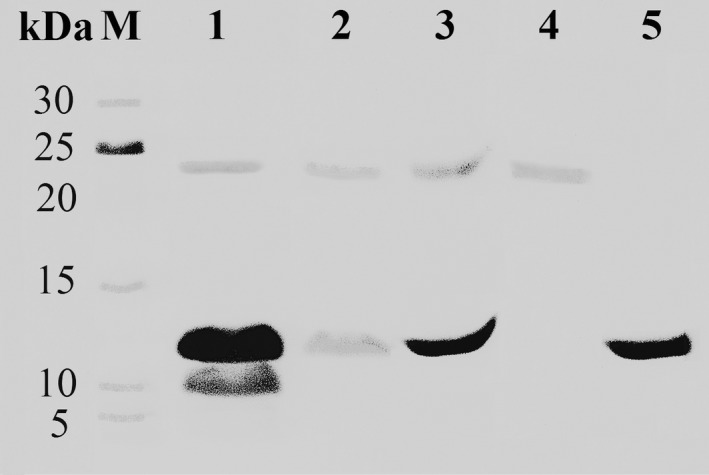
SDS/PAGE of balnase samples at different stages of its purification. 1 – chromatography on phosphocellulose P‐11, 2 – chromatography on UNOS6 column, 3 – chromatography on UNOS6 column after concentration and desalting, 4 – hypothetical protein HQ51_17865 of *B. altitudinis* after separation on UNOS_6_ column with step‐up profiles, 5 – balnase after separation on UNOS_6_ column with step‐up profiles.

**Figure 4:**
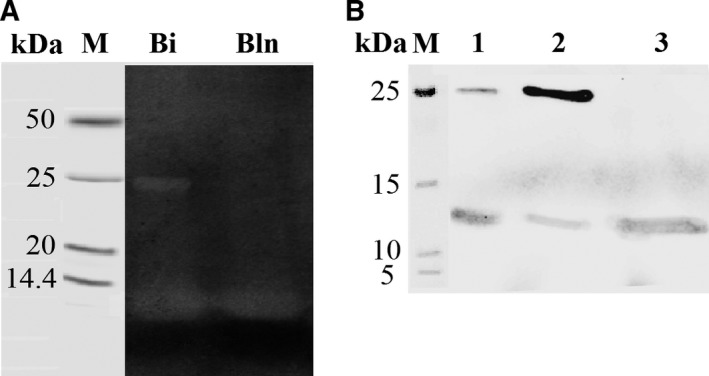
Zymography (A) and Western Blot analysis (B) of balnase sample after chromatography on UNOS6 column. 1, Bi – binase; 2 – chemically linked dimer of binase; 3, Bln – balnase fraction.

For the first step of routine RNase isolation we used two ion‐exchangers: a DEAE‐cellulose (anion‐exchanger), to get rid of negatively charged proteins, and a phosphocellulose P‐11 (cation‐exchanger), to concentrate the positively charged balnase. Using this technique, a protein sample with a yield of 75% (by activity) was obtained. The activity of the enzyme was 340 909 units/A_280_ (Table 1). The second step of purification was done using FPLC on the Biologic DuoFlow system. Proteins were eluted in 0.8 m NaCl (Fig. 2A). The profile of chromatographic peak had a slight asymmetry (Fig. 2A). To check the purity of the enzyme, we performed SDS/PAGE (Fig. 3). Protein separation on the gel revealed two bands with the molecular masses of 12 and 24 kDa (Fig. 3, lane 2, 3). To analyze the nature of the protein with the high molecular weight, we performed zymography analysis (Fig. 4A). It was shown that only 12 kDa protein had the RNase activity while the 24 kDa protein did not cleave the substrate (Fig. 4A, lane Bln). Using immunoblot analysis we revealed that anti‐binase antibodies interact with low molecular mass proteins only (Fig. 4B, lane 3). To identify these proteins we performed mass spectrometry analysis using the LC/MS‐MS system. It was shown that 12 kDa protein represents secreted RNase from *B. altitudinis* and 24 kDa protein is a hypothetical protein HQ51_17865 of *B. altitudinis* (data not shown).

**Table 1:** Isolation and purification of *Bacillus altitudinis* RNase

Stage of purification	Vol (V), mL	A_280_, units	Specific activity, units/A_280_	Degree of purification	Yield (by activity), %
Culture fluid after 28 h of cultivation	500	16	1250	1	100
After DEAE‐cellulose, pH 5.0	500	10	5000	68	98
After elution in 200 mm Na phosphate buffer, pH 7.0	20	1.1	340 909	273	75
After rechromatography on UNOS_6_ column using FPLC system	4	0.86	1.2 × 10^6^	960	40

A bioinformatics assay of these proteins revealed a high level of similarity in the physico‐chemical properties of balnase and the hypothetical protein (Table 2). This was the reason why both of them eluted together as one peak on ion‐exchange chromatography column (Fig. 2A).

**Table 2:** Physico‐chemical properties of balnase and hypothetical protein HQ51_17865 of *Bacillus altitudinis*

Protein	Molecular weight, Da	Theoretical pI	Instability index	Aliphatic index	Grand average of hydropathicity
Balnase	12181.6	9.52	27.25	79.72	−0.393
Hypothetical protein HQ51_17865	21316.8	9.59	20.75	80.30	−0.371

### Analysis of balnase homogeneity

**Figure 5:**
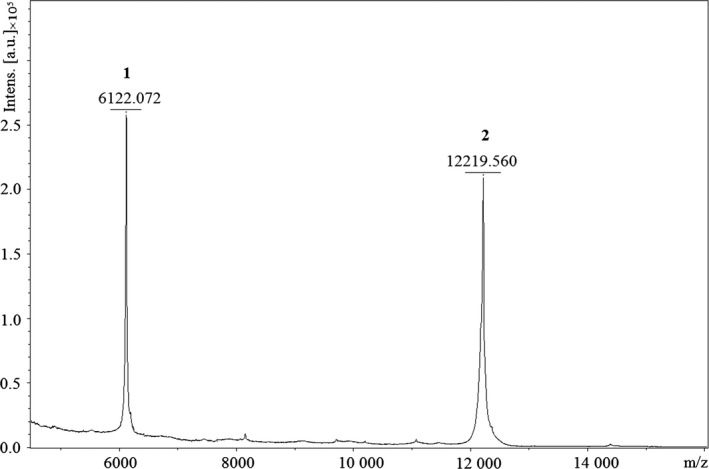
Mass spectrometry analysis of purified balnase sample after final FPLC chromatography with step‐up profiles. 1 – two‐charged ion of balnase, 2 – balnase.

To improve the resolution of enzyme peaks we used gradient elution with step‐up profiles and increased time for every stage of chromatography. This method allowed us to separate two proteins which were eluted at different salt concentrations. For balnase it was 0.3 m NaCl, and for the unknown protein it was 0.35 m NaCl (Fig. 2B). SDS/PAGE confirmed the separation of two proteins (Fig. 3, lane 4, 5). Moreover, it was shown that only balnase possesses RNase activity. The purity of RNase was checked by mass spectrometry analysis (Fig. 5).

So, the chromatography with step‐up profiles allowed us to obtain a homogeneous enzyme with the specific activity of 1.2 × 10^6^ units per 1 mg. The total yield of the balnase sample was 40% (Table 1).

## Discussion

Now, RNase from *B. thuringiensis* described by Dementiev 6 is named balnase due to the new taxonomic state of this microorganism. A bioinformatic search for a binase‐like gene in 12 completed genomes of *B. thuringiensis* species failed to find similar sequences. Further identification of the RNase‐producing strain was performed using modern mass spectrometry analysis as well as sequencing of 16S and *rpoB* genes (GenBank Accession numbers JX129389.1 and JX129391.1, correspondingly). The obtained results allowed us to rename the *B. thuringiensis* strain B‐388 as *B. altitudinis* B‐388.

The species *B. altitudinis* was discovered in air samples collected at an altitude of 41 km in Hyrabadad, India 28. The RNase secreted from *B. altitudinis*, balnase, is a close homolog of binase, which is known to possess antitumor and antiviral activities 2, 5 and 3. The primary structures of both RNases differ by one amino acid only. Nonpolar hydrophobic alanine at the position 106 in a balnase molecule is replaced by polar uncharged threonine in binase 6. It is interesting that the chemical properties of these amino acids are opposite. The hydroxyl group of threonine is fairly reactive, being able to form hydrogen bonds with a variety of polar substrates. Threonine can also be phosphorylated, and in the extracellular environment it can be O‐glycosylated 29. Alanine is one of the strongest helix formers (helix propensity 142), whereas threonine is one of the strongest β‐sheet formers (β‐sheet propensity 119) 30. This property of threonine is considered as crucial for amyloid fibril formation. In amyloidogenic proteins, this change can lead to interactions with another domain followed by self‐aggregation and can facilitate protein–protein interactions 31. Thus, there is ample evidence to demonstrate the importance of A to T substitutions in proteins of eukaryotic origin, that initiate an enhanced attention to A106T replacement reported for balnase as compared with binase 6 and 26.

The investigation of *B. altitudinis* growth revealed that balnase is secreted in the beginning of the growth retardation phase of bacterial growth with an accumulation peak at 28–34 h of cultivation (Fig. 1). Similar data were obtained for other extracellular RNases of bacilli 26, 27, 32.

Full protocol for routine RNase purification was reported by Dementiev *et al*. in 1993 6. The authors asserted that the method allowed for the isolation of the enzyme, which was purified 2228‐fold. The last stage of RNase purification was based on the principals of high‐performance reversed phase chromatography (reversed phase HPLC). The authors could not reach a high resolution of protein peaks that could indicate the presence of impurities in the sample. The homogeneity of balnase was confirmed by SDS/PAGE only 6. Reversed phase chromatography depends on the hydrophobic binding properties of the medium, the hydrophobicity of the solute and the composition of the mobile phase. Apparently, the balnase fraction obtained by Dementiev *et al*. contained unknown proteins with physico‐chemical properties similar to RNase. Using this method, we cannot reach the homogeneity of the balnase protein sample. Mass spectrometry analysis revealed the presence of different impurities in the protein preparation (data not shown). Therefore, we have optimized the protocol for balnase purification and suggested a new, modern technique for the rapid isolation of the homogeneous enzyme sample using ion‐exchange FPLC.

**Figure 6:**
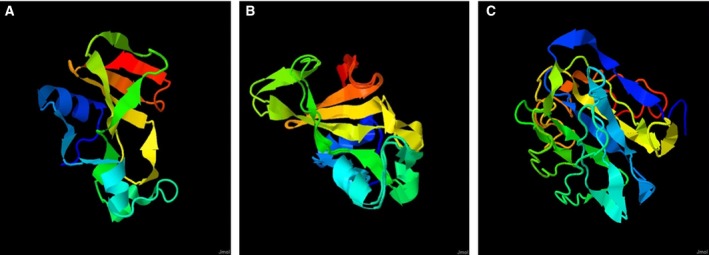
Putative models of balnase and hypothetical protein HQ51_17865 of *Bacillus altitudinis*. (A) – balnase, (B) – superposition of balnase and binase (PDB 1BUJ), (C) ‐ hypothetical protein HQ51_17865. The models of balnase and hypothetical protein were generated using their amino acid sequences on I‐Tasser server.

The first step of balnase purification we performed using DEAE‐cellulose and phosphocellulose P‐11. The activity of the enzyme at this stage was 340 909 units/A_280_ (Table 1). The ion‐exchange FPLC allowed us to increase the purity of the balnase sample (Fig. 2A and 3, lane 2, 3), however, the asymmetry of the peak profile and SDS/PAGE revealed the presence of two proteins in the eluate (Fig. 2A and 3). It is known that peak asymmetry could indicate the presence of different oligomeric forms of enzyme in the sample 33. Moreover, it was found that binase forms very stable dimers which do not dissociate during SDS/PAGE 25. These facts denoted the possibility of balnase to dimerize. This assumption was checked using zymography and immunoblotting analyses (Fig. 4). It was shown that a protein of high molecular weight does not possess RNase activity and the affinity for anti‐binase antibodies (Fig. 4). The obtained data indicated that balnase could not form stable dimers like binase. We carried out modelling of the balnase structure using I‐Tasser server (Fig. 6A) and compared it with the binase structure in solution (PDB 1BUJ) (Fig. 6B). It was shown that the two proteins have identical structures, with some differences in the Asp53 – Asn57 region (Fig. 6B). Probably, these changes influence the ability of balnase to form stable dimers. Mass spectrometry analysis identified a protein of high molecular weight as the hypothetical protein HQ51_17865 of *B. altitudinis* (data not shown).

The bioinformatics assay showed that the hypothetical protein has biochemical properties similar to balnase, which explained the same elution conditions of the two proteins using ion‐exchange chromatography (Table 2) and the low resolution of protein peaks described by Dementiev *et al*. 6. Using NCBI CDD and BLAST facilities, it was found that the hypothetical protein HQ51_17865 of *B. altitudinis* does not have conserved domains or any authentic similarities with previously studied proteins. To assess the possible functions of the hypothetical protein, its three‐dimensional structure was modelled using the I‐Tasser server (Fig. 6C). Functions of the hypothetical protein were predicted to deal with the participation in carbon and phosphorus exchanges. Probably, RNase and this protein are secreted together and participate in similar biological processes. Thus, the identical pI, aliphatic and instability index, and hydropathicity properties of the two proteins (Table 2) complicated their separation.

The removal of low‐level impurities has been facilitated by gradient elution with step‐up profiles (Fig. 2B). This approach allowed us to separate proteins and obtain a homogeneous sample in a process of three steps (Fig. 2B). The purity of the RNase sample was confirmed by highly sensitive mass spectrometry analysis (Fig. 5). Only two protein peaks were detected. One possesses the molecular weight around 12 kDa which corresponds to molecular weight of balnase. Another has half the mass and therefore represents two‐charged ion of balnase. The total yield of balnase was 40% by activity, with the specific activity of 1.2 × 10^6^ units (Table 1).

Thus, we have developed a rapid, modern technique for the purification of the new RNase from *B. altitudinis*. This method allowed us to obtain a homogeneous balnase protein in preparative amounts for its further investigation.

## Author contributions

Conceived and designed the experiments: VU, RS, VV, OI. Performed the experiments: ED, VK, LD. Analyzed the data: ED, VU, RS, VV, AK, OI. Contributed reagents/materials/analysis tools: UV, AK, OI. Wrote the paper: ED, VU, OI.
